# Correction: Discovery of dibenzylbutane lignan LCA derivatives as potent anti-inflammatory agents

**DOI:** 10.1039/d4md90023e

**Published:** 2024-06-17

**Authors:** Zhen Wang, Juan Zhang, Conghao Gai, Jing Wang, Xiaobin Zhuo, Yan Song, Yan Zou, Peichao Zhang, Guige Hou, Qingguo Meng, Qingjie Zhao, Xiaoyun Chai

**Affiliations:** a Department of Organic Chemistry, School of Pharmacy, Naval Medical University Shanghai 200433 China qjzhao_325@126.com chaixy1207@163.com; b School of Pharmacy, Key Laboratory of Molecular Pharmacology and Drug Evaluation, Yantai University Yantai 264005 China zhenwang0703@163.com qinggmeng@163.com; c Navy Medical Center, Naval Medical University Shanghai 200433 China; d School of Pharmacy, Binzhou Medical University Yantai 264003 China

## Abstract

Correction for ‘Discovery of dibenzylbutane lignan LCA derivatives as potent anti-inflammatory agents’ by Zhen Wang *et al.*, *RSC Med. Chem.*, 2024, https://doi.org/10.1039/d4md00053f.

There was an error in the structural configuration of the compound LCA and its derivatives shown in Fig. 1 and Scheme 1.

Fig. 1 should appear as follows:

**Fig. 1:**
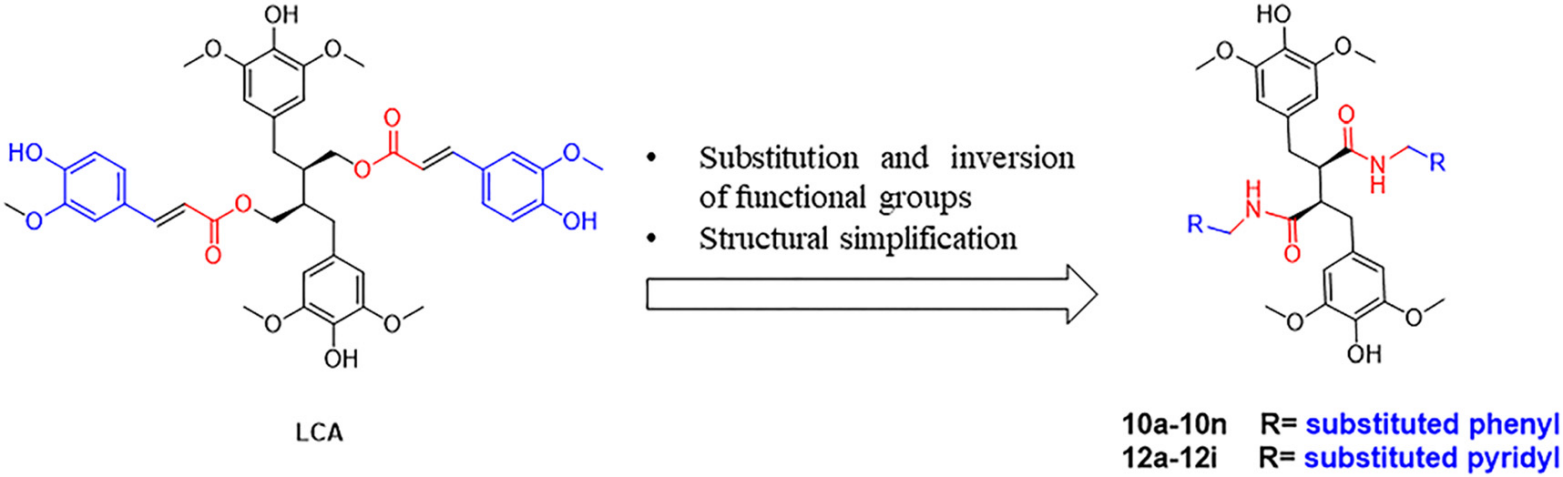
Design of target compounds.

Scheme 1 should appear as follows:

**Scheme 1:**
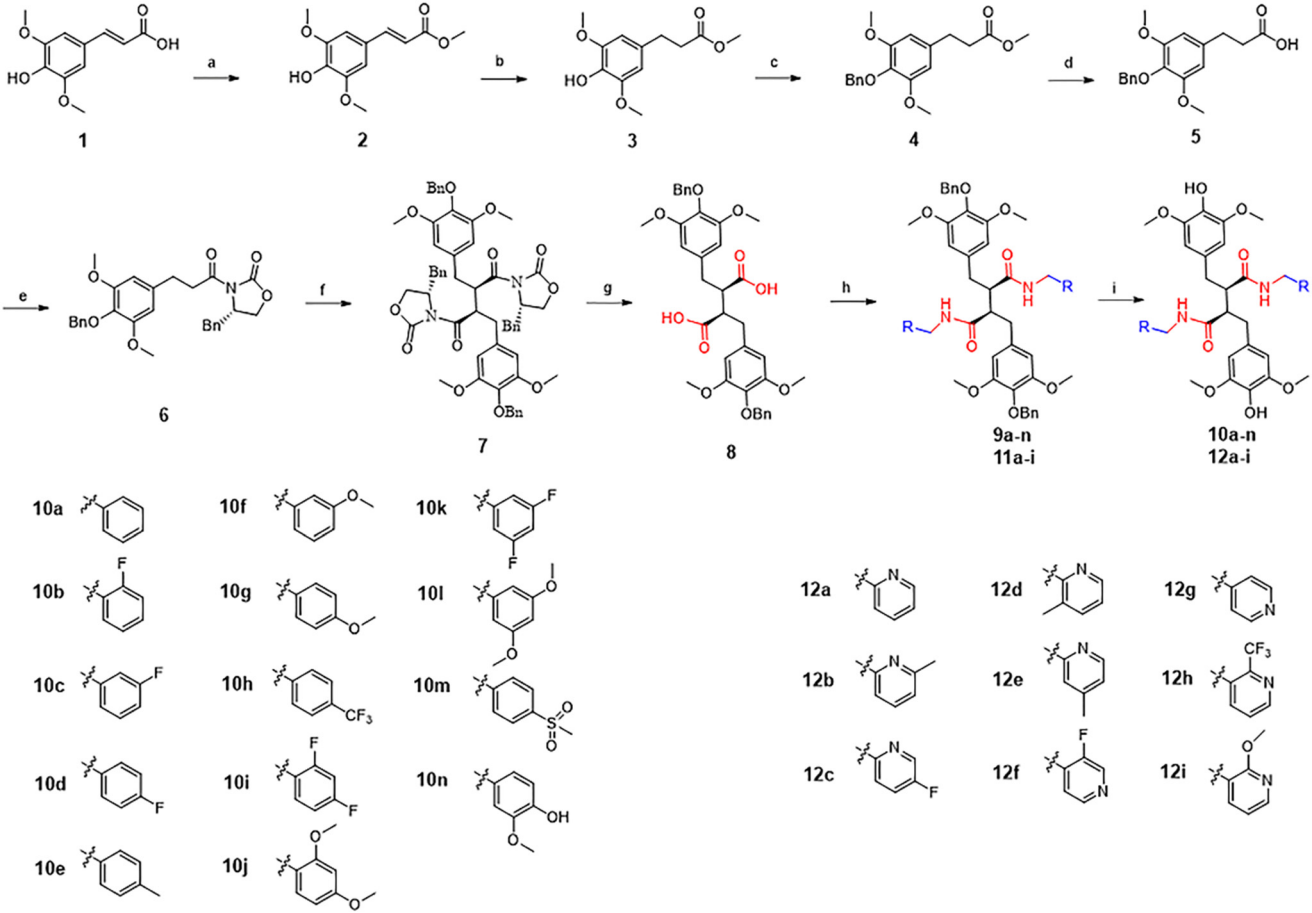
Synthesis of target compounds. Reagents and conditions: (a) sulfuric acid, MeOH, reflux, 5 h; (b) H_2_, Pd/C, MeOH, rt, 10 h; (c) benzyl bromide, K_2_CO_3_, acetone, reflux, 5 h; (d) NaOH, MeOH, reflux, 3 h; (e) (*S*)-4-benzyloxazolidin-2-one, *N*,*N*-dicyclohexylcarbodiimide, 4-dimethylaminopyridine, CH_2_Cl_2_, rt, 12 h; (f) lithium diisopropylamide, iodobenzene diacetate, tetrahydrofuran, −78 °C, 2 h; (g) lithium hydroxide monohydrate, H_2_O_2_, tetrahydrofuran, H_2_O, 0 °C, 15 min, then rt, 12 h; (h) *O*-benzotriazole-*N*,*N*,*N*′,*N*′-tetramethyl-uronium-hexafluoro-phosphate, *N*,*N*-diisopropylethylamine, *N*,*N*-dimethylformamide, rt, 4 h; (i) H_2_, Pd/C, MeOH, rt, 3 h.

The Royal Society of Chemistry apologises for these errors and any consequent inconvenience to authors and readers.

